# Prediction model for future OHCAs based on geospatial and demographic data: An observational study

**DOI:** 10.1097/MD.0000000000038070

**Published:** 2024-05-10

**Authors:** Kristian Bundgaard Ringgren, Vilde Ung, Thomas Alexander Gerds, Kristian Hay Kragholm, Peter Ascanius Jacobsen, Filip Lyng Lindgren, Anne Juul Grabmayr, Helle Collatz Christensen, Elisabeth Helen Anna Mills, Louise Kollander Jakobsen, Harman Yonis, Carolina Malta Hansen, Fredrik Folke, Freddy Lippert, Christian Torp-Pedersen

**Affiliations:** a Department of Anesthesia and Intensive Care, North Denmark Regional Hospital, Hjoerring, Denmark; b Department of Public Health, University of Copenhagen, København, Denmark; c Department of Cardiology, Aalborg University Hospital, Aalborg, Denmark; d Department of Respiratory Diseases, Aalborg University Hospital, Aalborg, Denmark; e Copenhagen Emergency Medical Services, University of Copenhagen, Copenhagen, Denmark; f National Clinical Registries, Frederiksberg, Denmark; g Department of Cardiology, Nordsjaellands Hospital, Hillerød, Denmark; h Department of Cardiology, Copenhagen University Hospital – Herlev and Gentofte, Herlev, Denmark; i Department of Clinical Medicine, University of Copenhagen, Copenhagen, Denmark; j Falck Danmark, Copenhagen, Denmark.

## Abstract

This study used demographic data in a novel prediction model to identify areas with high risk of out-of-hospital cardiac arrest (OHCA) in order to target prehospital preparedness. We combined data from the nationwide Danish Cardiac Arrest Registry with geographical- and demographic data on a hectare level. Hectares were classified in a hierarchy according to characteristics and pooled to square kilometers (km^2^). Historical OHCA incidence of each hectare group was supplemented with a predicted annual risk of at least 1 OHCA to ensure future applicability. We recorded 19,090 valid OHCAs during 2016 to 2019. The mean annual OHCA rate was highest in residential areas with no point of public interest and 100 to 1000 residents per hectare (9.7/year/km^2^) followed by pedestrian streets with multiple shops (5.8/year/km^2^), areas with no point of public interest and 50 to 100 residents (5.5/year/km^2^), and malls with a mean annual incidence per km^2^ of 4.6. Other high incidence areas were public transport stations, schools and areas without a point of public interest and 10 to 50 residents. These areas combined constitute 1496 km^2^ annually corresponding to 3.4% of the total area of Denmark and account for 65% of the OHCA incidence. Our prediction model confirms these areas to be of high risk and outperforms simple previous incidence in identifying future risk-sites. Two thirds of out-of-hospital cardiac arrests were identified in only 3.4% of the area of Denmark. This area was easily identified as having multiple residents or having airports, malls, pedestrian shopping streets or schools. This result has important implications for targeted intervention such as automatic defibrillators available to the public. Further, demographic information should be considered when implementing such interventions.

## 1. Introduction

Despite multiple initiatives to improve outlooks for out-of-hospital cardiac arrest (OHCA), 30-day survival remains relatively low at 10% in the US, 8% in Europe and around 16% in Denmark.^[1–4]^ The acute nature of OCHA and the critical time-dependency are 2 reasons why the survival chances are low; for each minute without cardio-pulmonary resuscitation (CPR) and defibrillation, the survival drops by 7% to 10%.^[5]^ The emergency medical services (EMS) has long recognized this, and the EMS response time has been a key parameter for benchmarking OHCA treatment for years. Nevertheless, it is difficult for any EMS system to provide timely response to all OHCAs.

Recognizing this as a potential limitation to improve survival, there has been a surge in activation of volunteer responders, especially in order to reach the bulk of OHCAs that happen in residential areas.^[6]^ These networks typically consist of local volunteers who are alerted by the emergency medical dispatch in case of a suspected OHCA. Volunteers are then either directed towards a publicly available external automated defibrillator (AED) and then to the site, or guided straight to the site to perform CPR.^[7]^

While CPR greatly increases the chance of survival after OHCA, a recent study found that with CPR alone, the chance of survival remains low if no further help arrives within 10 to 13 minutes.^[8]^ High-quality CPR, however, in combination with the use of an AED increases survival dramatically.^[9,10]^

Be it EMS, volunteer responder or publicly available AEDs, the key to a swift response is proximity. Proximity can be achieved either by increasing the number of responders, professionals as well as volunteers or increasing AED availability. Without proper prediction of future risk areas, proximity is either immensely resource heavy or unobtainable.

It is evident from previous research, that socioeconomic factors associated with OHCA incidence and outcome are strongly tied to geographical places of settlement, and it has previously been shown that age, immigration status and educational level are associated with an increase in incidence of OHCA.^[11–19]^ When trying to improve future effort, using retrospective geographical incidence is straightforward, but also a fragile approach. This paper combines data on previous incidence with a prediction model including socioeconomic factors, age, ethnicity and education on the most granular level possible to create a novel approach to target future area-targeted initiatives to improve OHCA preparedness in Denmark, and evaluates this approach.

## 2. Methods

### 2.1. Setting

Denmark provides an entirely tax-financed health care system, and thus the availability of services, including the EMS is free of charge, providing a formal equality of availability to all inhabitants of all socioeconomic layers.

### 2.2. Data collection

Every call that EMS dispatch suspects is an OHCA in Denmark triggers a response, and if any kind of resuscitative effort is initiated from either bystander, first-responder or EMS, the EMS personnel is obliged to fill out a form to the Danish Cardiac Arrest Registry. This is also the case, if the EMS dispatch did not initially suspect it to be an OHCA.

From this registry, we collected a Global Positioning System (GPS) coordinate of ambulance halt on site of the OHCA. Arrests without valid GPS were excluded. Data on population size, mean age and education-level for each hectare of Denmark were obtained through a private geodata company, relying on data from Statistics Denmark.^[20,21]^ From the same source, data regarding points of public interests in the form of airports, malls, schools, pedestrian streets w/multiple shops, public transport stations and major roads in the hectare was obtained. OHCAs within the same hectare were treated as multiple observations and merged with population data.

### 2.3. Permissions

This study complies with the Declaration of Helsinki and did not include human subjects. Data on previous OHCAs contained only GPS location and year, and demographic data was provided on a hectare level with no microdata enabling identification of individuals. The study was approved by the Danish Data Protection Agency. The Danish National Committee on Health Research Ethics does not require ethical approval for registry-based studies. The use of the Danish Cardiac Arrest Registry used for the conduct of this study was approved in the North Denmark Region (2008-58-0028).

### 2.4. Predictor variables

Alongside age, immigration status and educational level of the residents of the hectare, data available on points of public interest hypothesized to influence persons in the hectare, and thereby risk of an OHCA, were included as predictor variables. As such, points of public interest included hubs of transportation, both public transportation and private motoring. Furthermore, available data on public population centers exemplified by schools, malls and pedestrian streets with multiple shops were included as hypothesized areas with increased person-flow.

Each hectare was categorized in an area group according to characteristics that is either known or hypothesized to influence the OHCA incidence (Table 1). A single hectare could belong to different area groups over time and a hierarchical ordering was used to categorize the hectare of Denmark according to the first match in Table 1 starting with the first row. The hierarchy of points of public interest is shown in Table 1. Points of public interest were defined in 2019 and applied to previous years. If a hectare did not contain any point of public interest, it was categorized according to the number of residents and the workforce. Residents were defined as the number of people with a registered residential address in the hectare, stratified into a priori defined stratae with assumed external validity. Workforce was defined as the number of people employed on an address within the hectare.

**Table 1:** Variables and models used for prediction.

Area group	Predictor variables	Prediction model
Airport	None	Average incidence
Mall	None	Average incidence
School	Pedestrian street w/multiple shops, public transport station, major road, number of residents and workforce, age 60+, immigration from non-western country and lower educational level	Random forest (num. trees = 500)
Pedestrian street w/multiple shops	Public transport station, major road, number of residents and workforce, age 60+, immigration from non-western country and lower educational level	Random forest (num. trees = 500)
Public transport station	Major road, number of residents and workforce, age 60+, immigration from non-western country and lower educational level	Random forest (num. trees = 500)
Major road	Number of residents and workforce, age 60+, immigration from non-western country and lower educational level	Random forest (num. trees = 40)
No PoPI, 100 to 1000 residents	Workforce, age 60+, immigration from non-western country and lower educational level	Logistic regression
No PoPI, 50 to 100 residents	Workforce, age 60+, immigration from non-western country and lower educational level	Logistic regression
No PoPI, 10 to 50 residents	Workforce, age 60+, immigration from non-western country and lower educational level	Logistic regression
No PoPI, 0 to 10 residents	Workforce, age 60+, immigration from non-western country and lower educational level	Logistic regression
No PoPI, only workforce	Workforce	Logistic regression

PoPI = point of public interest.

For prediction, we aggregated the following characteristics of the population in each hectare: the proportion, as a number from 0 to 1, of the population aged 60 years or older, the proportion of non-western immigrants, and the proportion of the population with low education levels. Non-western immigrants included descendants of non-western immigrants and unknown origin, whereas lower educational level was defined as highest completed educational level being grade school, high school, vocational training or unknown educational level. Age, immigration status and educational level were categorical variables measured as a fraction of the residents in each category. Risk in airports was confined to the parts of the airport estimated to be buildings and entrances, whereas schools, which are also a mix of buildings and open areas, were included in their entirety, because open areas generally constitute a smaller fraction in comparison to airports. Hectares with no point of public interest, no residents and no workforce were analyzed separately. All predictor variables are listed in Table 1.

### 2.5. Statistical methods

We calculated the previous OHCA incidence rate according to the categories shown in Table 1 for each calendar year between 2016 and 2019, and calculated the mean OHCA incidence rate per km^2^ in each category across the calendar period.

We predicted the risk of at least 1 OHCA per year per hectare based on the data from 2016 to 2018 using the predictor variables and models shown in Table 1. The hectare-specific risk of at least 1 OHCA per year was predicted by using a random forest model in hectares with a point of public interest except for Mall and Airport, where no model was used, and the predicted risk was simply the average incidence in the years 2016 to 2018. The random forest model bootstrapts from the original dataset, makes a defined number of decision trees, as per Table 1, and aggregates the results. The approach was chosen under the assumption that they were special areas, uninfluenced by other categories in the hierarchy. Logistic regression models were used in hectares without a point of public interest (Table 1) where the number of covariates was less suited for random forest. For all random forest models, the number of trees used was 500, except for hectares with a major road, where for computational reasons we used only 40 trees. The logistic regression models included all variables shown in Table 1 and all possible interactions. All methods and covariates were chosen a priori by the researchers.

Using data from 2019 we compared the predicted risks of each hectare with the binary outcome (1 = at least 1 OHCA in 2019, 0 = no OHCA in 2019) using the mean squared error, known as the Brier score.^[22]^ To assess the predictive performance of our model we used a benchmark model which ignored the predictor variables and predicted the probability of at least 1 OHCA per year for each hectare by the average yearly incidence in that hectare in the training data (2016–2018). The benchmark model would correspond to assuming that areas of future incidence are the same as areas of previous incidence. The interpretation is that a lower Brier score in comparison is the superior statistical prediction model.

Predicted risk is calculated per hectare to ensure granularity for national, clinical utilization, and multiplied to report risk per km^2^ to facilitate external interpretation. This means that maximum risk is 100 instead of 1, and corresponds to 100% chance of an event. When presenting risk per km^2^ each km^2^ is comprised of 100 hectares which may not be adjacent.

All data from 2016 to 2019 were used to fit our final model from which we report the predicted risks of at least 1 OHCA per year per hectare. Data analysis was conducted using R statistical software version 4.1.1 with attached packages.^[23–31]^

Geographical representation was produced using QGIS.^[32]^

## 3. Results and discussion

### 3.1. Previous incidence

In the period 2016 to 2019, Denmark was divided into 4,345,831 hectares. Table 2 shows the distribution of area group and the incidence of OHCAs according to the hierarchical categorization (Table 1). Between 2016 and 2019 there were a total of 19,090 OHCAs with a valid GPS coordinate. 1240 OHCAs were excluded because of missing GPS data. Table 2 shows the percentage of total area and incidence, respectively, according to area type sorted by predicted risk. The red bars, representing the areas of highest predicted risk per km^2^ and the cyan bars, representing the areas of lowest predicted risk per km^2^ respectively, are accumulated in the pie charts. As is evident from Table 2, combining malls, public transport stations, pedestrian streets with multiple shops and areas with no point(s) of public interest but 10 to 50, 50 to 100, and 100 to 1000 residents respectively to account for 64.85% of OHCAs between 2016 and 2019 and only 3.4% of the area, corresponding to 1476 km^2^.

**Table 2:** Area and previous incidence according to area type and year.

Area group	Number	2016	2017	2018	2019	Mean incidence/km^2^	‍Area km² in % of total	^‍^OHCA incidence in % of total
Airport	km^2^	7	7	7	7	1.4	‍0.1	0.2‍
	OHCAs	12	8	11	9		‍	‍
Mall	km^2^	7	7	7	7	4.9	‍0.1	‍0.7
	OHCAs	32	41	28	29		‍	‍
School	km^2^	25	25	25	25	1.8	‍0.5	‍1.0
	OHCAs	42	50	55	36		‍	‍
Pedestrian street w/multiple shops	km^2^	16	16	16	16	5.8	0.3‍	‍2
	OHCAs	99	107	95	78		‍	‍
Public transport station	km^2^	5	5	5	5	3.0	0.1	‍0.3
	OHCAs	16	17	14	11		‍	‍
Major Road	km^2^	1321	1321	1321	1321	0.3	23.8	‍8.5
	OHCAs	419	399	423	380		‍	‍
No PoPI, 100–1000 residents	km^2^	41	42	35	44	9.7	0.7	‍8.2
	OHCAs	380	454	372	358		‍	‍
No PoPI, 50–100 residents	km^2^	79	79	73	82	5.5	1.4	‍9.1
	OHCAs	427	459	427	419		‍	‍
No PoPI, 10–50 residents	km^2^	1334	1343	1294	1347	1.7	23.9	‍48.7
	OHCAs	2333	2370	2327	2274		‍	‍
No PoPI, 0–10 residents	km^2^	2480	2476	2686	2503	0.3	45.6	‍17.8
	OHCAs	798	811	875	910		‍	‍
No PoPI, only workforce	km^2^	190	181	205	204	0.9	‍3.5	3.8‍
	OHCAs	162	158	235	165		‍	‍
No PoPI, no residents or workforce	km^2^	37,890	37,894	37,722	37,834	0.0	‍0.01	0‍
	OHCAs	319	271	317	273		‍	‍

Numbers are rounded from 2 decimals.

PoPI = point of public interest.

### 3.2. Predictive performance

We evaluated our model against the benchmark model, and the results are depicted in Table 3. As evident from this table, our prediction model performs better than the benchmark model in every area type.

**Table 3:** Predictive performance

Area group	Mean predicted risk per km²	Predicted risk per km² 95% CI	Benchmark Brier score	Our model Brier score
Airport	1.01	1.01:1.01	0.0019	0.0014
Mall	4.51	4.51:4.51	0.0466	0.0390
School	1.2	0:29.4	0.0173	0.0129
Pedestrian street w/multiple shops	5.45	0:47	0.0553	0.0425
Public transport Station	2.53	0:40.2	0.0327	0.0228
Major road	0.24	0:77.5	0.0035	0.0028
No PoPI, 100–1000 residents	8.45	2.39:85.85	0.0811	0.0641
No PoPI, 50–100 residents	5.02	0.91:81.14	0.0554	0.0444
No PoPI, 10–50 residents	1.69	0.06:85.96	0.0203	0.0156
No PoPI, 0–10 residents	0.34	0:41.17	0.0045	0.0035
No PoPI, only workforce	0.88	0.85:80.69	0.0090	0.0076

PoPI = point of public interest.

### 3.3. Predicted incidence

The areas with the highest predicted mean annual risk per km² of one or more OHCAs occuring were areas with no point of public interest and 100 to 1000 residents (8.45[2.39:85.85]), pedestrian street with multiple shops (5.45[0.00:47.0]), areas with no point of public interest and 50 to 100 residents (5.02[0.91:81.14]) and malls (4.51[4.5:4.5]). Public transport stations and areas of no point of public interest and 10 to 50 residents represent other areas of elevated mean predicted annual risk with 2.53(0.0:40.2) and 1.69 (0.06:86) respectively. Predicted annual risks are depicted in Table 3.

**Figure 1.**
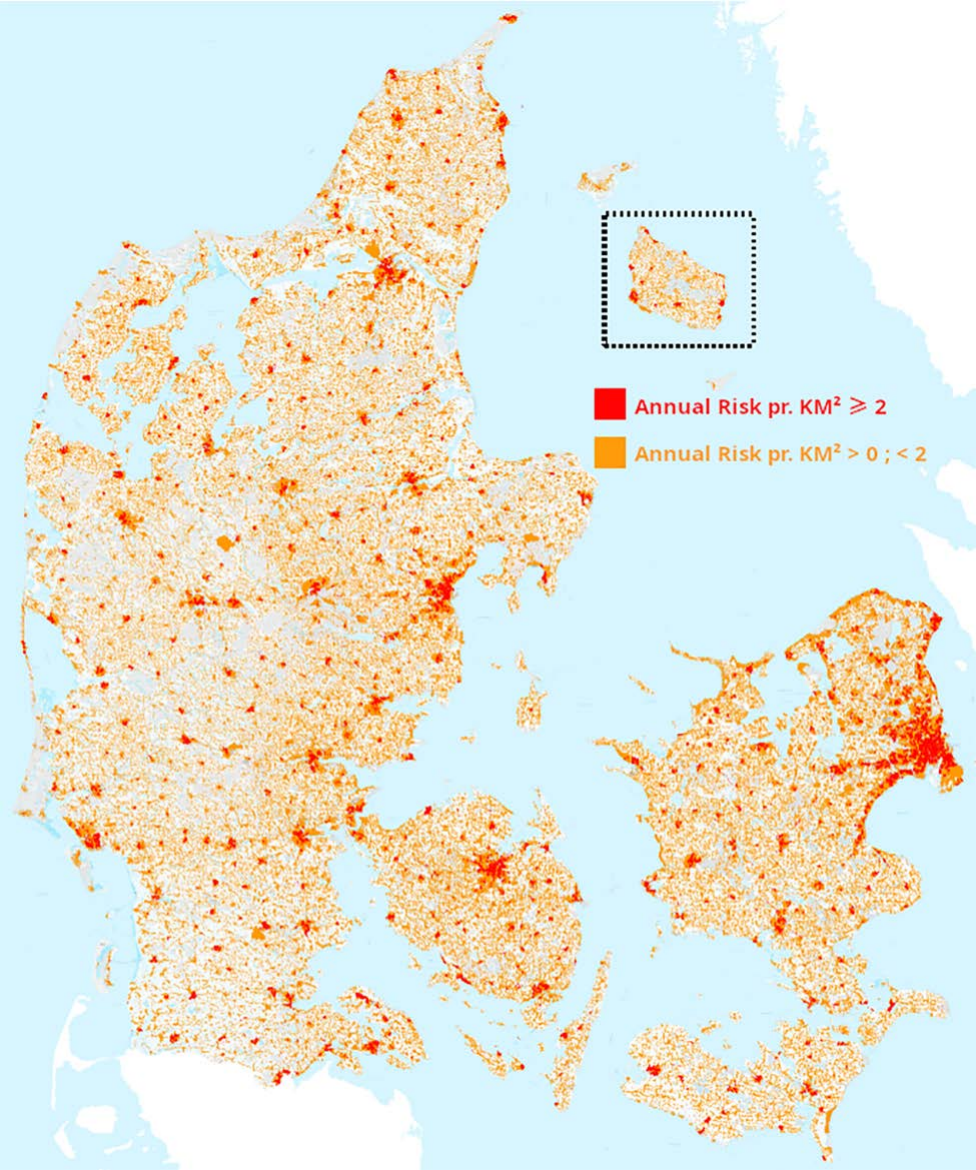
Geographical representation of predicted risk of OHCA. Predicted annual risk of at least 1 OHCA is plotted as orange for annual risk per km² between 0 and 2, both excluded, and red for 2 and above. Hectares with no color have no data for prediction or a risk of 0. OHCA = out of hospital cardiac arrest.

The individual predicted annual risk of one or more OHCA for each hectare was stratified into 2 groups, 0 to 2 both excluded, and ≥2 and plotted on a map of Denmark as shown in Figure 1.

## 4. Discussion

This study identifies previous OHCA incidence as being concentrated to geographically small areas, and demonstrates that a prediction model including population characteristics is superior when identifying areas of future incidence.

Previous OHCA incidence is consolidated in selected points of public interest and areas with a high resident population density. While the 95% confidence intervals for risk per km^2^ are broad, it is clear from Table 3 that the prediction model is better at identifying areas where the next OHCA might happen than by simply looking at areas of previous arrests. When looking at the geography in the prespecified categories, the previous incidence and the prediction model align in determining high-incidence categories. The prediction model provides an individual risk for each hectare, of which the mean is presented above. While both previous incidence and predicted risk is high in relation to areas covered by certain points of public interest, the vast majority of OHCAs happen in the, geographically larger, areas of dense population but without points of public interest such as malls or pedestrian streets with multiple shops.

In relation to previous studies of geo-spatial analyses of OHCA, our data benefits from having one national registry for all OHCAs and a very high level of capture and quality of OHCA data. Obtaining data on geographical and population parameters on a national scale is rare, but ultilizing such data is not without caveats. Differences in data availability across countries may limit the external value of present studies, as the available data points chosen in this study is defined by a national entity, and might be differently defined, or altogether unobtainable in other countries. Other, internationally acknowledged standards of geographical division, such as the “Degree of Urbanisation” (DEGRUBA) would allow for more external validity of present study, but would have decreased granularity tremendously, and would be too inaccurate to utilize in terms of AED placement, as coverage of such are few hundred meters.^[33]^ Further, only selected parts of the Danish geography is covered by the DEGRUBA division. Previous attempts to analyze incidence and outcome of OHCA incidence have largely focused on the characteristics of the individual suffering an OHCA, rather than the geographical characteristics of the site; thus making it less suitable for geographical analysis and prediction. A previous study of geographical data has shown the importance of age and socioeconomic status, but did not consider population density which, in the current study, turns out to be of pivotal importance.^[34]^ Another study on geography of OHCA using a methodology similar to our but with a more modest amount of includes arrests, only used age and sex as demographic predictors, and made predictions on a coarser, municipality-level scale.^[35]^

EMS response time has had much attention, as it is one of the key factors in improving outcome after OHCA.^[36–40]^ While we have valid data on the OHCAs included, the EMS response time is subject to great changes from incident to incident depending on where the nearest unit is at any given time. As the amount of rural areas with some risk of OHCA is so immense, relying only on EMS response in all of these areas will be unrealistic, this is already part of the motive for implementing a widespread system of professional- and volunteer responders.^[41]^

While according to this study, most points of public interest have a slightly increased risk, the risk is particularly high in malls, pedestrian streets with multiple shops, and public transport stations. Even after adjustment for areas with people, the predicted risk in airports remained low when compared to other points of public interest. Knowing that Denmark’s largest airport, has a yearly incidence over 7 cardiac arrests, this airport alone is considered to account for the vast majority of total cardiac arrests at airports, and given that military airports were included due to data limitations, the actual risk of specific airports might be underestimated.^[42]^ Preparedness in airports is known to be very good in terms of CPR rate and AED accessibility.^[42]^

When planning future efforts, it is important to bear in mind that the majority of the incidence is located in residential areas. This is a field of great opportunity for improvement, as only 3.8% to 6.4% of the OHCAs occurring in private residences in the period were defibrillated prior to EMS arrival.^[2]^ It has previously been concluded that focusing AED placement on public places rather than residential areas would be more beneficial due a relatively higher frequency of shockable rhythm in witnessed OHCAs.^[43]^ It is, however, uncertain whether this finding is due to delay in AED application in residential areas, and the results in this paper points towards AED placement in selected residential areas with a dense population as a very important supplement to areas of special activities as above. Previous studies have proposed a coverage radius of a publicly available AED to a quarter mile (~400 m).^[44]^ This corresponds to an area of around 0.5 km^2^. If applied to our findings, that almost 65% of OHCAs occur within an area of 1476 km^2^ a total of only 2952 well-placed AEDs would theoretically provide coverage of the areas of highest predicted risk. At present there are well over 20,000 publicly available AEDs in Denmark.^[45]^ When instead considering each hectare separately, as per Figure 1, only 55,116 hectares corresponding to 511 km^2^ have predicted annual risk above the threshold of 2 per km^2^. Future research and intervention should ensure that, at the very least, these areas are covered by an AED.

These findings are very specific to the Danish geography, however, the importance of the selected area types, and the notion to include both points of public interest and demographic data when making geographic predictions of future OHCAs are universal.

### 4.1. Limitations

Firstly, no matter how good a prediction model is, it comes with inaccuracy inherent in all attempts to predict the future. Therefore, this study does not claim to produce certainties, but makes a probable guess on possible future occurrences.

Secondly, only OHCAs where a resuscitative effort is initiated are recorded, and arrests in very remote areas may not be discovered until resuscitation is futile, leading to a possible underestimation of incidence in rural areas. These arrests, however, would not constitute an area of potential improvement, as dispatch of AEDs, volunteer responder programs and EMS relies on timely Acknowledgments of OHCA.

Third, the study is, to an extend, defined by data availability, which both limited the amount of freedom in study design and might limit the potential for external reproduction.

## 5. Conclusion

The great majority of OHCAs in Denmark occurred in a very small geographical area of Denmark. Most occurred in residential areas with a high density of residents, followed by public streets with commerce. Prediction models including demographic data seems to be efficient for identification of high risk areas and targeted intervention.

## Author contributions

**Conceptualization:** Kristian Bundgaard Ringgren, Thomas Alexander Gerds, Christian Torp-Pedersen.

**Data curation:** Kristian Bundgaard Ringgren, Helle Collatz Christensen, Harman Yonis.

**Formal analysis:** Vilde Ung, Thomas Alexander Gerds.

**Funding acquisition:** Christian Torp-Pedersen.

**Methodology:** Kristian Bundgaard Ringgren, Thomas Alexander Gerds, Kristian Hay Kragholm, Christian Torp-Pedersen.

**Project administration:** Kristian Bundgaard Ringgren, Christian Torp-Pedersen.

**Resources:** Freddy Lippert.

**Software:** Kristian Bundgaard Ringgren, Vilde Ung, Thomas Alexander Gerds, Peter Ascanius Jacobsen, Filip Lyng Lindgren, Anne Juul Grabmayr.

**Supervision:** Thomas Alexander Gerds, Kristian Hay Kragholm, Peter Ascanius Jacobsen, Filip Lyng Lindgren, Anne Juul Grabmayr, Helle Collatz Christensen, Louise Kollander Jakobsen, Carolina Malta Hansen, Fredrik Folke, Freddy Lippert, Christian Torp-Pedersen.

**Validation:** Kristian Bundgaard Ringgren, Vilde Ung, Thomas Alexander Gerds, Freddy Lippert.

**Visualization:** Kristian Bundgaard Ringgren, Peter Ascanius Jacobsen, Filip Lyng Lindgren.

**Writing – original draft:** Kristian Bundgaard Ringgren.

**Writing – review & editing:** Kristian Bundgaard Ringgren, Vilde Ung, Thomas Alexander Gerds, Kristian Hay Kragholm, Peter Ascanius Jacobsen, Filip Lyng Lindgren, Anne Juul Grabmayr, Helle Collatz Christensen, Elisabeth Helen Anna Mills, Louise Kollander Jakobsen, Harman Yonis, Carolina Malta Hansen, Fredrik Folke, Freddy Lippert, Christian Torp-Pedersen.

## References

[1] Jensen TW Blomberg SN Folke F. The National Danish cardiac arrest registry for out-of-hospital cardiac arrest – a registry in transformation. Clin Epidemiol. 2022;14:949–57. 35966902 10.2147/CLEP.S374788 PMC9374329

[2] Ringgren KB Christensen HC Schønau L. Danish cardiac arrest registry – English summary [cited May 6, 2021]. Available at: https://hjertestopregister.dk/?page_id=428.

[3] Sudden Cardiac Arrest Foundation. [cited 2021 Feb 4]. Latest AHA statistics on cardiac arrest survival reveal little progress. Available at: https://www.sca-aware.org/sca-news/latest-aha-statistics-on-cardiac-arrest-survival-reveal-little-progress.

[4] Gräsner JT Wnent J Herlitz J. Survival after out-of-hospital cardiac arrest in Europe - Results of the EuReCa TWO study. Resuscitation. 2020;148:218–26. 32027980 10.1016/j.resuscitation.2019.12.042

[5] Larsen MP Eisenberg MS Cummins RO Hallstrom AP. Predicting survival from out-of-hospital cardiac arrest: a graphic model. Ann Emerg Med. 1993;22:1652–8. 8214853 10.1016/s0196-0644(05)81302-2

[6] Oving I Masterson S Tjelmeland IBM. First-response treatment after out-of-hospital cardiac arrest: a survey of current practices across 29 countries in Europe. Scand J Trauma Resusc Emerg Med. 2019;27:112. 31842928 10.1186/s13049-019-0689-0 PMC6916130

[7] Valeriano A Van Heer S de Champlain F Brooks SC. Crowdsourcing to save lives: a scoping review of bystander alert technologies for out-of-hospital cardiac arrest. Resuscitation. 2021;158:94–121. 33188832 10.1016/j.resuscitation.2020.10.035

[8] Shahzleen R Mads W Fredrik F. Association of bystander cardiopulmonary resuscitation and survival according to ambulance response times after out-of-hospital cardiac arrest. Circulation. 2016;134:2095–104. 27881566 10.1161/CIRCULATIONAHA.116.024400

[9] Valenzuela TD Roe DJ Nichol G Clark LL Spaite DW Hardman RG. Outcomes of rapid defibrillation by security officers after cardiac arrest in casinos. N Engl J Med. 2000;343:1206–9. 11071670 10.1056/NEJM200010263431701

[10] Bækgaard JS. Viereck S Møller TP Ersbøll AK Lippert F Folke F. The effects of public access defibrillation on survival after out-of-hospital cardiac arrest. Circulation. 2017;136:954–65. 28687709 10.1161/CIRCULATIONAHA.117.029067

[11] Hui I Cho WKT. 3.13 – Spatial dimensions of american politics. In: Huang B, ed. Comprehensive Geographic Information Systems. Oxford: Elsevier; 2018. p. 181–8.

[12] Massey DS. American apartheid: segregation and the making of the underclass. AJS. 1990;96:329–57.

[13] Lockwood T Coffee NT Rossini P Niyonsenga T McGreal S. Does where you live influence your socio-economic status? Land Use Policy. 2018;72:152–60.

[14] Anderson LM Charles J S Fullilove MT Scrimshaw SC Fielding JE Normand J. Providing affordable family housing and reducing residential segregation by income: a systematic review. Am J Prev Med. 2003;24(3, Supplement):47–67. 12668198 10.1016/s0749-3797(02)00656-6

[15] White MJ Kim AH. Residential segregation. In: Kempf-Leonard K, ed. Encyclopedia of Social Measurement. New York: Elsevier; 2005. p. 403–9.

[16] Jonsson M Ljungman P Härkönen J. Relationship between socioeconomic status and incidence of out-of-hospital cardiac arrest is dependent on age. J Epidemiol Community Health. 2020;74:726–31. 32385129 10.1136/jech-2019-213296 PMC7577091

[17] van Nieuwenhuizen BP Oving I Kunst AE. Socio-economic differences in incidence, bystander cardiopulmonary resuscitation and survival from out-of-hospital cardiac arrest: a systematic review. Resuscitation. 2019;141:44–62. 31199944 10.1016/j.resuscitation.2019.05.018

[18] Allan KS Ray JG Gozdyra P. High risk neighbourhoods: the effect of neighbourhood level factors on cardiac arrest incidence. Resuscitation. 2020;149:100–8. 32068027 10.1016/j.resuscitation.2020.02.002

[19] Raun LH Jefferson LS Persse D Ensor KB. Geospatial analysis for targeting out-of-hospital cardiac arrest intervention. Am J Prev Med. 2013;45:137–42. 23867019 10.1016/j.amepre.2013.03.013

[20] Statistics Denmark. [cited February 6, 2024]. Available at: https://www.dst.dk/en/.

[21] Viamap - Specialist in visualizing data on maps. 2022 [cited February 6, 2024]. Available at: https://www.viamap.net/en/.

[22] Gerds & Kattan. Medical Risk Prediction Models. Chapman & Hall; 2021:312.

[23] R Core Team. 2021. R. Vienna, Austria: R Foundation for Statistical Computing; 2021. Available at: https://www.R-project.org/.

[24] R Studio Team. RStudio: Integrated Development Environment for R. RStudio, PBC, Boston, MA. 2020.

[25] Hadley W. R package “ggplot2”. Springer-Verlag New York; 2016. Available at: https://ggplot2.tidyverse.org.

[26] Hadley W Evan M. R package “haven”. 2021. Available at: https://CRAN.R-project.org/package=haven.

[27] Rytgaard HC Anders M Torp-Pedersen C. R package “heaven”. 2021. Available at: https://github.com/tagteam/heaven.

[28] Frank EH Jr, with contributions from Charles Dupont and many others. R package “Hmisc”. 2021. Available at: https://CRAN.R-project.org/package=Hmisc.

[29] Matt D Arun S. R package “data.table”. 2021. Available at: https://CRAN.R-project.org/package=data.table.

[30] Pebesma EJ Bivand RS. R package “sp”. 2005. Available at: https://cran.r-project.org/doc/Rnews/.

[31] Roger B Tim K Barry R. R package “rgdal”. 2021. Available at: https://CRAN.R-project.org/package=rgdal.

[32] QGIS Development Team. QGIS Geographic Information System. Open Source Geospatial Foundation; 2021. Available at: https://qgis.org.

[33] Degree of Urbanisation (DEGURBA) — European Environment Agency. [cited February 5, 2024]. Available at: https://www.eea.europa.eu/data-and-maps/data/external/degree-of-urbanisation-degurba.

[34] Straney LD Bray JE Beck B Bernard S Lijovic M Smith K. Are sociodemographic characteristics associated with spatial variation in the incidence of OHCA and bystander CPR rates? A population-based observational study in Victoria, Australia. BMJ Open. 2016;6:e012434. 10.1136/bmjopen-2016-012434 PMC5129006 27821597

[35] Auricchio A Peluso S Caputo ML. Spatio-temporal prediction model of out-of-hospital cardiac arrest: designation of medical priorities and estimation of human resources requirement. PLoS One. 2020;15:e0238067. 32866165 10.1371/journal.pone.0238067 PMC7458314

[36] Grunau B Kawano T Scheuermeyer F. Early advanced life support attendance is associated with improved survival and neurologic outcomes after non-traumatic out-of-hospital cardiac arrest in a tiered prehospital response system. Resuscitation. 2019;135:137–44. 30576783 10.1016/j.resuscitation.2018.12.003

[37] Yamaguchi Y Woodin JA Gibo K Zive DM Daya MR. Improvements in Out-of-Hospital Cardiac Arrest Survival from 1998 to 2013. Prehosp Emerg Care. 2017;21:616–27. 28426258 10.1080/10903127.2017.1308604

[38] Mathiesen WT Bjørshol CA Kvaløy JT Søreide E. Effects of modifiable prehospital factors on survival after out-of-hospital cardiac arrest in rural versus urban areas. Crit Care. 2018;22:1–9. 29669574 10.1186/s13054-018-2017-x PMC5907488

[39] Holmén J Herlitz J Ricksten SE. Shortening ambulance response time increases survival in out-of-hospital cardiac arrest. J Am Heart Assoc. 2020;9:e017048. 33107394 10.1161/JAHA.120.017048 PMC7763420

[40] Bürger A Wnent J Bohn A. The Effect of ambulance response time on survival following out-of-hospital cardiac arrest. Deutsches Arzteblatt international. 2018;115:541–8. 30189973 10.3238/arztebl.2018.0541 PMC6156551

[41] Andelius L Malta Hansen C Lippert FK. Smartphone activation of citizen responders to facilitate defibrillation in out-of-hospital cardiac arrest. J Am Coll Cardiol. 2020;76:43–53. 32616162 10.1016/j.jacc.2020.04.073

[42] Gantzel Nielsen C Andelius LC Hansen CM. Bystander interventions and survival following out-of-hospital cardiac arrest at Copenhagen International Airport. Resuscitation. 2021;162:381–7. 33577965 10.1016/j.resuscitation.2021.01.039

[43] Weisfeldt ML Everson-Stewart S Sitlani C. Ventricular tachyarrhythmias after cardiac arrest in public versus at home. N Engl J Med. 2011;364:313–21. 21268723 10.1056/NEJMoa1010663 PMC3062845

[44] Srinivasan S Salerno J Hajari H Weiss LS Salcido DD. Modeling a novel hypothetical use of postal collection boxes as automated external defibrillator access points. Resuscitation. 2017;120:26–30. 28847755 10.1016/j.resuscitation.2017.08.220 PMC5730075

[45] Tal og fakta om hjertestart. [cited April 3, 2024]. Available at: https://hjertestarter.dk/find-hjertestartere/danmark-er-i-verdensklassen-naar-det-gaelder-genoplivning (only available in Danish.

